# Pharmaceutical company perspectives on current safety risk communications in Japan

**DOI:** 10.1186/2193-1801-3-51

**Published:** 2014-01-24

**Authors:** Hisashi Urushihara, Gen Kobashi, Hideaki Masuda, Setsuko Taneichi, Michiko Yamamoto, Takeo Nakayama, Koji Kawakami, Tsutomu Matsuda, Kaori Ohta, Hiroki Sugimori

**Affiliations:** Division of Drug Development and Regulatory Science, Faculty of Pharmacy, Keio University, Shiba-koen, Minato-ku, Tokyo, 105-8512 Japan; Department of Planning and Management, National Institute of Radiological Sciences, Anagawa, Inage-ku, Chiba, 263-8555 Japan; Healthcare Communications, Communication Design Division, Dentsu Public Relations Inc, Tsukiji, Chuo-ku, Tokyo, 104-0045 Japan; Laboratory of Health Education, Graduate School of Education, The University of Tokyo, Hongo, Bunkyo-ku, Tokyo, 113-0033 Japan; Education Centre for Clinical Pharmacy Practice, Showa Pharmaceutical University, Higashi-Tamagawagakuen, Machida, Tokyo, 194-8543 Japan; Department of Health Informatics, Graduate School of Medicine and Public Health, Kyoto University, Yoshidakonoe-cho, Sakyo-ku, Kyoto, 606-8501 Japan; Department of Pharmacoepidemiology, Graduate School of Medicine and Public Health, Kyoto University, Yoshidakonoe-cho, Sakyo-ku, Kyoto, 606-8501 Japan; Department of Pharmaceutical and Medical Device Regulatory Science, Graduate School of Medical Science, Yamagata University, Iida-Nishi, Yamagata, Yamagata, 990-9585 Japan; Research Center for Charged Particle Therapy, National Institute of Radiological Sciences, Anagawa, Inage-ku, Chiba, 263-8555 Japan; Department of Preventive Medicine, Graduate School of Sports and Health Science, Daito Bunka University, 560 Iwadono, Higashi-Matsuyama-shi, Saitama, 355-8501 Japan

**Keywords:** Drug risk, Safety communication, Drug company, Regulation, Questionnaire survey

## Abstract

In 1987, a group infection of hepatitis in patients receiving a contaminated fibrinogen product was first reported to the Japanese regulatory agency. Eventually, this serious drug incident involved more than 10,000 cases of infection. In response, the Government of Japan established a responding inspection committee in 2008 to make recommendations for the restructuring of drug regulatory administration. The final report was issued in 2010. One agenda item of this restructuring was the improvement of drug-related safety risk communications. Our research group on drug safety risk communications, which is funded by the Government of Japan, surveyed pharmaceutical companies regarding their perspective on current risk communications. The survey was conducted using an anonymous questionnaire developed for this study which included the three operational domains of targets, contents, and measures of drug risk communication. Fifty-two of the 74 member companies of the Post-marketing Surveillance Subcommittee of the Japan Pharmaceutical Manufacturer’s Association participated, and this response rate of more than 70% was considered sufficient to ensure the external validity of the survey results. Results showed that the most highly prioritized aspect of risk messaging was the strength of evidence, and that outcome evaluation of risk communication gained recognition. Further, while physicians and pharmacists were the most prioritized communication targets, pharmacovigilance departments devoted the most resources to regulators, at more than 30%. The Internet was recognized as a useful public source of risk information, whereas Drug Guides for Patients delivered on the web were considered under-recognized. Further discussion of these results with the aim of enhancing the restructuring of the Japanese drug regulatory administration system are warranted.

**Electronic supplementary material:**

The online version of this article (doi:10.1186/2193-1801-3-51) contains supplementary material, which is available to authorized users.

## Background

Safety issues related to medical products have serious and significant implications not only for individual health, but occasionally also for public health on a large scale. Given the inherently uncertain safety and effectiveness of medicinal products, discussions of the importance and significance of benefit/risk communications with healthcare professionals and patients have been repeated, not only in the wake of particular drug disasters but on a routine basis (Bahri and Harrison-Woolrych [Bibr CR3][Bibr CR4]; Avorn 2008; Committee on Regulatory Restructuring for Inspection and Recurrence Prevention of Drug-Induced Hepatitis Disaster Case 2010; European Medicines Agency 2009). The Erice Declaration on Communicating Drug Safety Information in September 1997 asserted that risk communication is a public health activity which depends on the collective responsibility of all parties, involving patients and healthcare professionals, as well as multiple other stakeholders, including pharmaceutical companies, drug regulators, academia, researchers, media and others (The Uppsala Monitoring Centre 1997).

Pharmaceutical industries play a significant role in the safety risk communication of their medicinal products. They are the upstream supplier of information related to medicinal products, and at the same time the product supplier also. They are legally bound and socially responsible for the collection and evaluation of information related to the effectiveness, safety and rational use of medicinal products before and after market launch, and for the provision and necessary exchange of relevant information to healthcare professionals and patients, with the aim of optimizing the benefit-risk balance in the real world. Pharmaceutical companies consequently possess the most abundant information regarding the efficacy and safety of their products, and multiple communication techniques and channels, albeit that these are under strict regulatory supervision. In this communication flow, safety risk information for drugs sourced from pharmaceutical companies streams down to concerned regulators, healthcare professionals, patients and other parties of interest. Discussion primarily occurs between regulatory agencies and pharmaceutical companies to determine how, to whom, and by what measures safety risk information should be communicated. Despite strong recommendations for the tailoring of communications toward specific targets, however, there is scant opportunity for lay people, patients and practitioners to be involved in this discussion (Bahri and Harrison-Woolrych 2012b; European Medicines Agency 2013; Bahri 2010). Additionally, despite the fact that drugs require administration into patients’ bodies under the direction of practitioners to exert their efficacy, transparency is somewhat compromised for both patients and healthcare professionals with regard to the discussion over which risk information for medicinal products should be made publicly available, and when this information should be communicated, which in turn leads to public distrust to pharmaceutical companies (Olsen and Whalen 2009). The necessity of systematic investigation of safety risk communications carried out as part of the pharmacovigilance activities of pharmaceutical companies has long been recognized, but the number of reports appearing to date is markedly low (Giampaolo 2010; Ingate et al. 2006).

In the aftermath of a catastrophic drug disaster in Japan in which more than 10,000 patients treated with contaminated pharmaceutical products of fibrinogen and coagulation factor IX were infected with viral hepatitis (Horiuchi et al. 2009), the investigating committee’s final report in 2010 offered recommendations requiring the restructuring of the entire regulatory system for pharmaceutical products (Committee on Regulatory Restructuring for Inspection and Recurrence Prevention of Drug-Induced Hepatitis Disaster Case 2010). This report proposed the mobilization of information technology and involvement of patients in improved communications for drug safety within the Japanese post-marketing pharmacovigilance system.

In the present study, this government-funded research group on drug safety risk communication surveyed pharmaceutical companies for their views and perspectives on safety risk communication in Japan. The findings were expected to serve as a basis for ongoing discussion on this restructuring.

## Methods

### Study design

A questionnaire survey was conducted among persons in charge of the pharmacovigilance departments of member companies of the Japan Pharmaceutical Manufacturer’s Association (JPMA). The JPMA is the major industry association for pharmaceutical companies located in Japan, and all companies involved in the research and development of new medicinal products for human treatment and diagnosis are members.

### Survey questionnaire development

An anonymous, self-administered questionnaire was developed by a multidisciplinary study panel consisting of physicians, epidemiologists, a media expert, and a pharmacoepidemiologist with business experience in pharmacovigilance activities at a pharmaceutical company. In this survey, safety risk communication was defined as the exchange of drug information regarding safety risks by pharmaceutical companies with the aim of ensuring the rational use of drugs in practical clinical settings. Three operational domains related to safety risk communication were identified, namely contents, targets, and measures, and question items for each were developed by expert discussion. The questionnaire also enquired about background information of companies and respondents. The draft questionnaire was reviewed for face and content validities before finalization by an independent senior pharmacovigilance executive at a pharmaceutical company, and by the chairman of the Post-Marketing Surveillance (PMS) Subcommittee of the Drug Evaluation Committee of the JPMA. The final contents of the survey questionnaire were also reviewed and approved by the Subcommittee Chairman and by the Subcommittee Secretariat Officer regarding the protection of anonymity and confidentiality (Additional file 1).

### Questionnaire items

The contents domain included three items. The first item assessed the prioritization of eight important aspects of risk information, known as the Media Doctor Australia rating criteria for the adverse effects of media coverage of new medical treatments (Additional file 2) (Media Doctor Australia 2012). The other two items were open questions which asked about what factors are taken into consideration during the creation and conveyance of risk communication messages. The targets domain included three items regarding eight communication targets, namely patients, the public, physicians, pharmacists, paramedics, regulators, the media, and in-company divisions as reference. The evaluation included the relative importance of target audience and the degree of success of communication with the target audience. Further, respondents were also asked to estimate workload allocation for the individual target audiences within the pharmacovigilance department. The measures domain included 10 items. Drug Guides for Patients (DGPs) are created by the market authorization holders of drugs for three types of drugs, namely those with a Warning section in the package insert; those with a package insert requiring specific instructions to patients to prevent serious adverse drug reactions; and those requiring the provision of specific information to patients to ensure rational use, excluding drugs used for tests and surgeries (Pharmaceuticals and Food Safety Bureau 2005). This requirement for DGPs is similar to that for the Medication Guides prepared as a part of the US Risk Evaluation and Mitigation Systems (REMS) (The US Food and Drug Administration 2011). The DGPs were assessed according to the 10 rating criteria used in a survey of US Food and Drug Administration (FDA) publications on the internet conducted by the Center for Drug Evaluation and Research (CDER) (The US Food and Drug Administration 2009). Items included “easy to read”, “easy to find what I need”, “organization of information”, “font and font size”, “length”, “relevance to your specialty or area of expertise”, “understandable”, “timeliness”, “helpful”, and “amount of background information”. Rating was done on 5-point Likert scales from poor through average to excellent, with 3 as the mid-point and higher scores indicating a better result. Two items asked respondents to assess internet websites providing drug information which were operated by pharmaceutical companies themselves with regard to their usefulness for risk communications to patients and healthcare professionals. Further, respondents were also asked to choose the three most useful measures for risk communications in emergencies from among regulatory measures, including a DHCP letter and revision of the package insert; direct communication by the regulatory authority; communication by company medical representatives; publication via mass media or academic journals; and communication via company websites. They were also asked to rank these measures for effectiveness from first to third rank, with the first-ranked measure given a score of three and the third-ranked a score of one, while measures which were not selected were scored as zero. Last, we solicited opinions about disclosure of the risk management plan, for which a new local regulatory guidance had been issued (April 2012) but which had yet to be implemented at the time of survey (Pharmaceuticals and Food Safety Bureau 2012).

### Study procedure and analysis

Agreement on the objectives and protocol procedures was obtained from the JPMA PMS Subcommittee during study planning. The JPMA PMS Subcommittee office then sent the questionnaires with cover letters requesting participation via the postal service in mid-February 2011 to its 74 subcommittee member companies, independently from the study team to maintain survey respondent anonymity. The cover letter explained the outlines and objectives of the study, and mentioned the protection of respondent anonymity and confidentiality in disclosure to academic publications. Further, we asked that the respondent to the questionnaire should be a permanent employee(s) who was engaged in pharmacovigilance activities and was capable of representing the responding company’s opinions. The JPMA PMS Subcommittee office sent two reminder e-mails to the addressees, at the time the questionnaire was sent and 2 months later. The completed questionnaires were returned by postal mail directly to the study team. “No response” to a questionnaire item were excluded from the analysis, except for background information. The responses were summarized with descriptive statistics using SPSS ver 19 (IBM Corp., Armonk, NY, USA).

## Results

Fifty-two questionnaires were collected between mid-February 2011 and the end of April 2011 (return rate: 70.3%). After exclusion of one questionnaire which had more than half of its items unanswered, 51 questionnaires were eligible for analysis, of which 12 were from foreign capital companies (23.5%) and 34 from large-scale companies with more than a thousand permanent employees (66.7%). In 92.2% of the responding companies, employees engaged in pharmacovigilance activities accounted for fewer than 10% of all permanent employees. Forty-one respondents were supervisors (80.4%), and more than half were aged in their 50s (51.0%), followed by those in their 40 s (41.2%). Years of experience in post-marketing pharmacovigilance activities ranged 1 to 35, with a median of 11. Nineteen were the designated safety management supervisor under the Japanese Good Vigilance Practice Ministry Ordinance.

### Contents

**Figure 1:**
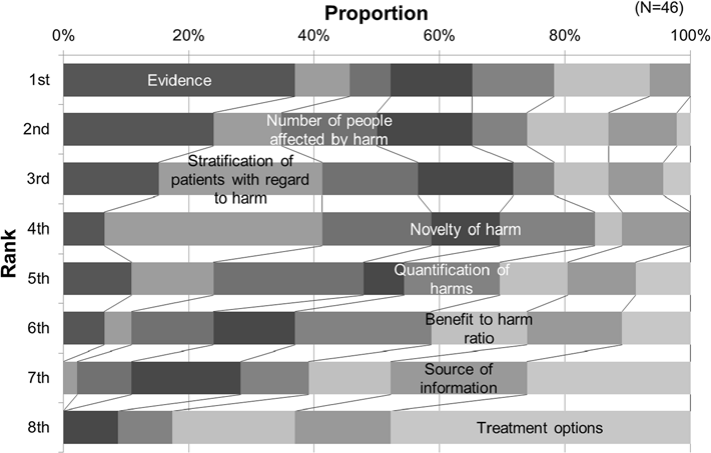
**Prioritization of aspects according to the criteria of Media Doctor Australia in risk communication messages for pharmaceutical products by pharmacovigilance staff at drug companies.** Evidence: Where relevant there is mention of the strength of evidence and correct interpretation. Stratification of patients with regard to harm: Mentions which groups of patients are most likely to be harmed. Number of people affected by harm: Some quantification of the number of people or percent of people affected by the harm. Novelty of harm: Mentions whether or not the harm was previously identified or mentions what is added to previous knowledge about it. Quantification of harms: Some quantification of harm in terms of severity. Benefit to harm ratio: Tries to balance reporting of both benefits and harms or gives some sense of the ratio between the two. Sources of information: Provides details on information sources and their potential COI, and reports independent source or mentions unsuccessful attempts to obtain corroboration. Treatment options: Mentions alternatives and discusses whether alternatives are more or less harmful. Each criterion was rated from first to eighth according to its importance in transmitting risk information.

The relative importance of eight aspects in the transmission of messages for risk information, which is known as the Media Doctor Australia rating criteria for adverse effects, was rated (Figure 1). Seventeen respondents ranked ‘evidence (mention of strength of evidence and correct interpretation)’ as the first-ranked aspect, followed by ‘stratification of patients with regard to harm (mentions which groups of patients are most likely to be harmed)’ and ‘number of people affected by harm (some quantification of the number of people or percent of people affected by the harm)’, with the total of the above three aspects accounting for approximately one-half of the first through fourth ranks. Less importance was given to the other aspects, with ‘treatment option’ assigned the lowest importance. When respondents were asked about what they paid attention to when creating messages for risk communication, 26 answered they would give consideration to the comprehensibility of contents, while 12 others focused on the prevention of health hazard as an outcome of risk communication. When asked what they would pay attention to in transmitting risk messages, 16 respondents answered the promptness of communication and 10 answered the medium used for communication. Four respondents emphasized the importance of behavioral changes as an outcome of message transmissions with the intention of risk minimization.

### Targets

**Figure 2:**
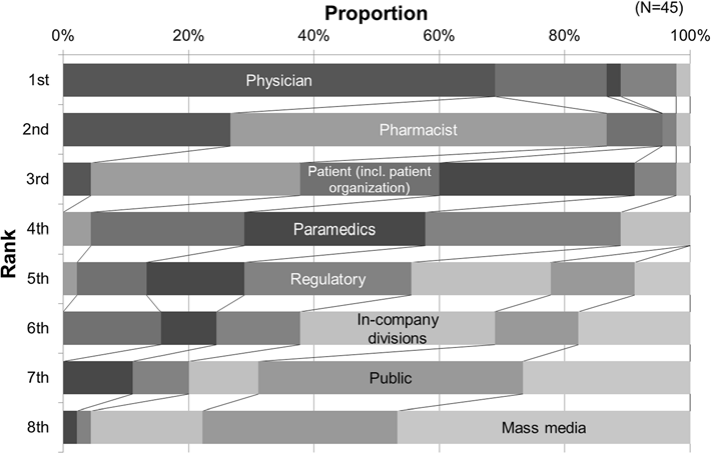
**Prioritization of target audiences by pharmacovigilance staff at pharmaceutical companies in safety risk communication for pharmaceutical products.** Respondents rated these criteria from first to eighth according to their importance as a target for risk communications.

**Figure 3:**
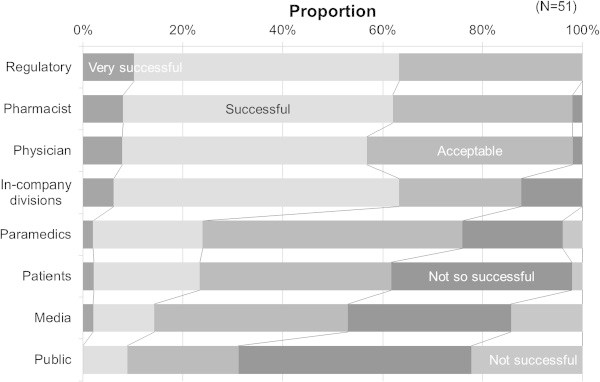
**Evaluation of success by pharmacovigilance staff at pharmaceutical companies concerning risk communications with concerned parties.**

**Figure 4:**
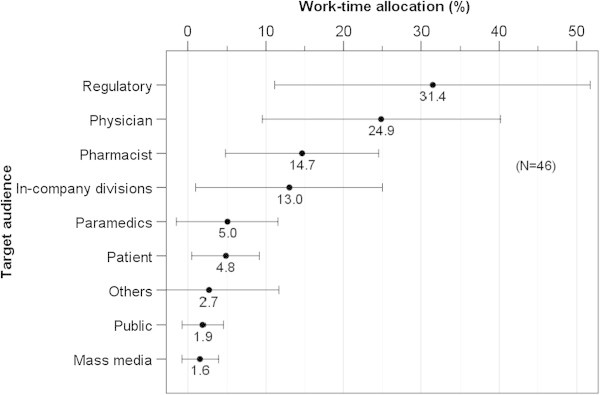
**Estimates of work-time allocation according to communication target by the pharmacovigilance departments of pharmaceutical companies. Presented in mean ± SD.**

Relative importance of the eight communication targets was rated (Figure 2). Physicians were ranked first by 31 respondents. Pharmacists were not ranked first by any respondent but were ranked second by 27 respondents; patients were ranked first by 8 respondents, second by 4, and third by 10; paramedics were ranked third by 14 respondents; and the regulatory authority was ranked fourth by 14. The public and media ranked lower than in-company divisions. The degree of subjective success in risk communication was assessed for each of the above communication parties (Figure 3). Risk communication with the regulatory agency was rated as most successful among the parties (‘fairly successful’ or above: 100%), followed by pharmacists and physicians (both 98.0%); whereas paramedics (76.0%), patients (61.7%), the media (53.1%) and the public (31.1%) were rated lower than in-company divisions (87.8%). Pharmacovigilance departments allocated the greatest time resources to the regulatory agency (mean 31.4%), followed by physicians (24.9%) and pharmacists (14.7%, Figure 4). The rough estimation of work-time allocation to paramedics (5.0%), patients (4.8%), the public (1.9%), and the media (1.6%) were smaller than that for in-company divisions (13.0%).

### Measures

**Figure 5:**
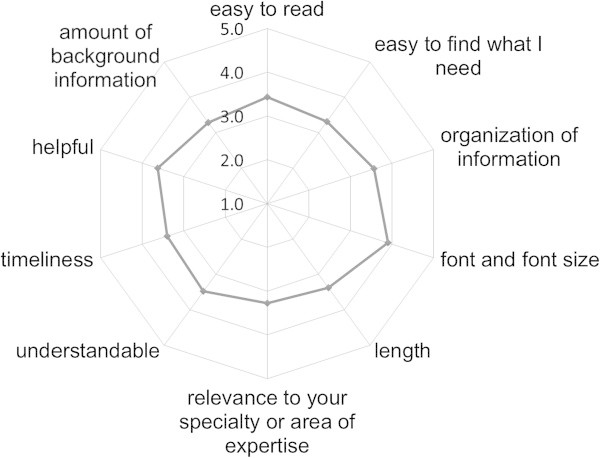
**Evaluation of Drug Guide for Patients by pharmacovigilance staff at pharmaceutical companies (n = 52).**

Forty-three respondents had experience in the creation of DGPs (84.3%). These were rated good for “font and font size” (mean score: 3.9), “helpful” (3.6), and “organization of information” (3.6), and 70% of the respondents evaluated them as useful for patients (Figure 5). However, nine respondents commented that DGPs are likely not utilized effectively by patients due to their low recognition among the public. Some commented that since all package inserts and corresponding DGPs are now accessible via the same PMDA homepage, the purpose and significance of DGPs, whose source information is encompassed in the package inserts, were questionable. 62.0% of respondents answered that it was useful to create a DGP for all newly approved drugs, particularly to ensure the disclosure of information, whereas 16 respondents (8.0%) answered that it would be sufficient to prepare a DGP for medicines requiring particular attention in use by patients and are administered by patients themselves, in line with the current regulations. New development of an additional tool for patients regarding revision of the ‘Precautions’ section of package inserts was considered unnecessary by more respondents (30.6%) than those who considered it necessary (24.5%). A respondent commented that such revisions should be incorporated into the DGP in order to preserve a single communication tool for patients.

62.0% of respondents assessed the websites run by pharmaceutical companies as effective for risk communication with patients and 76.0% assessed them as effective for risk communication with healthcare professionals, in contrast to low marks for usability given to the PMDA website in the comments. Advantages of these private websites included their rich contents and customizable design fitted for the purpose and intention of the companies, as well as their openness, easy and quick access, and interactivity. Major concerns were expressed regarding the limited range of patients with the tools and ability to access these sites, the varying levels of literacy among patients, biased or partial contents provided on these sites, and concerns related to the use of internet websites arising from the present legal prohibition against direct-to-consumer advertising for drugs. Of note, a few respondents commented that these sites provide an alternative, out-of-office hours communication tool for healthcare professionals, by complementing the office hour activities of medical representatives.

The respondents assessed the effectiveness of the eight channels for communicating risk information in emergency situations. Regulatory measures such as Dear Healthcare Professional letters and revisions of the package insert, direct communication by the regulatory authority, and communication by company medical representatives were rated as most effective (mean rating ± SD: 1.5 ± 1.1, 1.5 ± 1.2, 1.5 ± 1.2, respectively), followed by publication through the media (0.9 ± 1.1) and communication via company websites (0.4 ± 0.8).

67.3% of respondents considered that disclosure of the safety risk management plan was useful, with the expectation that this would make it easier to gain the cooperation of healthcare professionals and patients in ensuring effective implementation, and by reason of the public significance of ensuring the transparency of post-marketing activities. Concern was expressed over the potential of explicit scientific language to provoke unnecessary misunderstanding and unrest among patients, and over the need to tailor the language in such disclosures for lay people.

### Other comments

Among the numerous comments, notable responses included the need to establish a single integrated mechanism to communicate drug information to healthcare professionals and patients; the need to consider benefit-risk balance and the accurate comprehension of risks during risk communication; the expectation that the media should be a responsible party not only in always-negative but also positive involvement with responsibility in public risk communication; and the need for official guidelines and a regulatory department specialized in direct communications with healthcare professionals, taking into account the seriousness of the risk.

## Discussion

Here, we comprehensively investigated drug company perspectives on current risk communications for drug safety. To our knowledge, this is the first such study conducted in Japan.

The response ratio of 70% was considered sufficient to ensure the external validity of the survey results, and was additionally strengthened by the following considerations. More than 10 of the targeted companies are considered to be currently inactive or to have reduced post-marketing pharmacovigilance activities because their products are generic, legacy, or licensed out to other companies, and accordingly might not have responded to the survey. Foreign capital companies account for 25.6% of all member companies of the JPMA (Japan Pharmaceutical Manufacturers Association 2013), which is consistent with the ratio of domestic to foreign capital among the firms responding to this survey. Responses were predominately from large-scale companies with more than one thousand regular employees, which account for the majority of pharmaceutical companies selling newly developed pharmaceutical products in Japan. Many respondents appeared to be sufficiently experienced in the area of pharmacovigilance to provide adequate answers.

### Contents

To be successful, messages for risk communications need to convey a balance of multiple conflicting requirements: they need to be written in a clear, simple and comprehensive manner on topics that are relevant and important, and yet at the same time they need to be brief (Seligman and Osborne 2009; Mazor et al. 2005). We found that many companies prioritized the strength of evidence and place a high value on comprehension, probably because of their intent to ensure the credibility of the message. This appears to reflect the principle that the effectiveness of risk communication depends on the creditability of the message (Bahri and Harrison-Woolrych 2012b). Aspects at the next ranking level, ‘Stratification of patients with regard to harm’ and ‘Number of people affected by harm’, likely include the expectation of behavioral changes in medical practice as a consequence of the communication. This question lacked an option of ‘mention of what should be done differently’ since the Media Doctor rating criteria lack it; nevertheless, the comments of several respondents regarding the question on the creation of messages emphasized the importance of the outcome of message transmission, indicating that pharmaceutical companies are likely to next focus on the effectiveness of message transmission. The EU and US guidelines clearly recommend that communication plans include the assessment of effectiveness, such as quantification of behavioral changes, survey of comprehension, and reductions in adverse event reporting (Edwards and Chakraborty 2012; The US Food and Drug Administration 2009; European Medicines Agency 2013). The Japanese regulations for the provision of DHCP letters, in contrast, requires a pharmaceutical company to assesses only the distribution of the communication materials to medical institutions and practitioners in the communication plan. Unlike the EU and US plans, therefore, the Japanese communication plan does not address ‘true effectiveness’ (Pharmaceuticals and Food Safety Bureau 2011a). The introduction of risk management planning in Japan, although delayed until April 2013, urges the Japanese regulatory community to change the poor methodology of the current method of assessing communication practices (Pharmaceuticals and Food Safety Bureau 2012).

### Targets

Stakeholders and the message to be communicated in risk communications vary depending on the type of harm/risk. Communication with the most prioritized targets, physicians and pharmacists, was considered mostly successful, indicating that drug companies focused intensively on these two occupations, as evidenced by the considerable time allocated to them shown in the other question item. Patients were rated as third; however, risk communication with patients was rated as less successful than that with the reference in-company division in this survey. Drug companies in Japan communicate with patients in an indirect and unidirectional fashion via healthcare professionals, patient-oriented handouts, and corporate websites. Direct-to-consumer advertisements are legally prohibited, and drug company activities and publications directed to patients are strictly regulated. Given the comparatively small work-time allocation to patients, the high prioritization given to patients as communication targets in this survey therefore likely represents corporate policies and conceptual but unsubstantial patient-oriented attitudes.

We found the lower ranking of the regulatory authority as a communication target to be inconsistent with the finding that the regulatory authority received the largest work-time allocation. Pharmaceutical companies are required to consult the regulatory authority when commencing regulatory actions involving risk communication activities which target healthcare professionals and the public. We speculate that the drug companies may be concerned about being seen as excessively authority-oriented, with a view to subsequent publication of the survey results. Additionally, risk communications with the regulatory authority were all rated successful or better. We speculate that respondents might have censored their responses by selecting the socially “harmless” answer ‘successful’ to telegraph that they had no particular concerns in their relationship with the regulatory authority.

### Measures

Generally, DGPs obtained a good appraisal, particularly in terms of readability and contents. As respondents pointed out, however, they are poorly recognized by the public; in a survey of 1,707 people who had undergone a regular physical examination, for example, only 2% had experienced accessing a DGP and only 15% were in fact actually aware of them, and an initiative to attract patient attention to this material is therefore warranted (Suka 2011). Consistent with the US medication guides, the scope of drugs requiring the preparation of DPGs is limited (The US Food and Drug Administration 2011). However, more than half of respondents supported extension of the preparation of DGPs to all prescription drugs, as does the UK Patient Information Leaflet (Committee on Safety of Medicines 2005). In Japan, prescription drugs are dispensed with a consumer medication information (CMI) sheet, which includes a small picture of the drug in its dosage form and a brief description of its usage, dosage and adverse reactions. These are prepared by the dispensing pharmacy, which primarily sources them from non-authorized drug information databases maintained by private claims reimbursement computer system vendors. In this regard, a survey of the US CMI sheets reported that the contents were of low quality, and urged their improvement (Kimberlin and Winterstein 2008; Raynor et al. 2007). In contrast, DGPs provide several pages of rich and detailed content for patient self-instruction and are developed by the supplying pharmaceutical company and reviewed by the regulatory authority at the expense of national insurance subscribers and patients, although many are currently in disuse. Utilization of DGPs as legitimate source documents for the preparation of CMIs at dispensing pharmacies is strongly recommended. Achieving this will require that all information in individual DGPs for all prescription drugs become publicly available through an official online database with easy accessibility using the Standard Generalized Markup Language.

Our survey respondents also positively noted the efficiency and effectiveness of drug information websites run by the pharmaceutical companies. Although some respondents expressed concerned that the information posted on company-run websites is likely to be partial and one-sided (Davis et al. 2007), one study reported that lay people considered that the credibility of information on regulatory agency and pharmaceutical company websites was comparable (Kim 2011). The low usability of the PMDA website noted in our survey appears to result from its tangled, multiroute, multistep path from the home page to target information, and from the awkwardly organized, overlapping information and PDF regulatory documents attached to many webpages. This should be improved by usability testing from the perspective of the public, without influence by the regulatory perspective (Bahri and Harrison-Woolrych 2012b; European Medicines Agency 2013). The significance and importance of internet use in searching and querying drug information was shown in our separate surveys, which found that 37.4% of community pharmacists had utilized company-run websites and that 43% of people who received regular health checkups had had a chance to browse for drug information on the Internet (Suka 2011). The very major importance of official, integrated, and strategic web–based provision of drug information should therefore be acknowledged. The EMEA websites may be referenced as sophisticated examples, and the EU is also planning to establish a single consolidated online database for drug information (European Medicines Agency 2013).

The respondents appreciated the significance of direct communication of risk information by the regulatory agency, especially in case of emergency. Current Japanese regulatory advisories, including revision of label information, issuance of DHCP letters, and other safety notifications often require intensive and sometimes contentious preliminary discussion, followed by tough negotiation before agreement between the regulatory authority and pharmaceutical company is reached. These likely represent the most resource-consuming tasks on both sides. The risk communication measures implemented by drug companies under the strict oversight of the regulatory agency may therefore take considerable time until the dispatch of key risk messages can proceed, which might in turn impair the timeliness of risk mitigation actions, and also compromise the effectiveness and transparency of communications. Indeed, in July 2011, coincidentally after the close of this survey, the PMDA launched a new website “On information concerning the risks of drugs under evaluation” within its “Pharmaceuticals and Medical Devices Information Homepage”, similar to the Drug Safety Communication by the US FDA (Pharmaceuticals and Food Safety Bureau 2011b; Seligman and Osborne 2009). Further effective utilization of direct and rapid communication by the regulatory agency is desirable.

The media were recognized as and expected to play an essential role in emergency risk communications by the pharmaceutical company side. This expectation was in contrast to the lower success rating for communications targeted to mass media, and the smaller resource allocation to them. The effectiveness of risk minimization activities such as DHCP letters is reportedly influenced by whether the risk receives wide media publicity (Weatherby et al. 2001; Urushihara et al. 2011; Waller et al. 2006). However, the legitimate role of the media in risk communications has been questioned as their interests differ from those of pharmaceutical companies and regulatory agencies. If the media is conceived of as a public organ of society, rather than a private institution that seeks mere demagoguery and sensation, then its provision of well-balanced, evidence-based media coverage of safety issues may be expected to maximize benefits and ameliorate harms for patients and the public, although admittedly this remains an ongoing challenge (Bahri 2010; Waller et al. 2006). The advantage of the UK’s scientific media in improving the quality of reporting of drug information via the general media should be acknowledged (Czarnecki 2008; Mebane 2005).

Safety risk management planning was eventually implemented in April 2013 in Japan, following the issuance of local guidance for risk management plans for pharmaceuticals in April 2012, which supplements the ICH E2E “pharmacovigilance planning” guideline (Pharmaceuticals and Food Safety Bureau 2012). Most global companies have therefore already experienced the development and implementation of safety risk management plans, whereas domestic companies likely have little or no such experience. More than half of the respondents in this survey agreed with disclosure of the outlines of risk management plans, mostly owing to a likely grudging acceptance of transparency. Disclosure of safety risk management plans is planned to commence in 2013 and is also expected to facilitate understanding and cooperation with practitioners and patients. However, as our respondents noted, tailoring the information to the expected audience is essential, given its potential to both maximize the effectiveness of the information and minimize anticipated confusion among patients as well as healthcare professionals (Bahri and Harrison-Woolrych 2012b).

### Limitations

The results of this survey likely represent the partial and one-sided views of the responding pharmaceutical companies, and comprehensive and impartial investigations among other important stakeholders should accordingly follow. Owing to the anonymity of the study process, it was not possible to identify non-responding companies and thereby determine the extent of selection bias. The possibility exists that only those companies which actively express their opinion on a routine basis dominated this survey results. This survey did not target generic drug makers since they generally rely on the marketing authorization holders of the original drugs and do not play a central role in risk communication activities. Further, the answers derived from the survey may be biased toward those considered socially desirable, out of concern of criticism after the publication of this survey and subsequent to internal review within companies before the answers were returned. Even considering the above limitations, the high return rate appears sufficient to ensure representativeness in Japan, although the results would not be applicable to companies located in other countries, which have different medicosocial national systems.

## Conclusions

We conducted a survey to better understand current risk communications by drug companies located in Japan. Risk communications operate at two levels, to the individual patient and to the general public. For both levels, to be successful implementation, credible messaging is necessary not only about risks, but also the benefit-risk balance of the particular drug as well as its outcome evaluation. This notion appears to be well accepted by the pharmaceutical industry in Japan. Direct communication by the regulatory agency and mobilization of mass media channels may enhance the speed and effectiveness of emergency risk communications, but the utilization and quality control of these communications appear premature and should be further explored. Internet websites are heavily utilized to provide risk information by regulatory agencies, pharmaceutical companies and other concerned parties, but the current provision of information on drug safety is highly disparate, occurring in various forms at multiple websites. Establishment of a comprehensive, integrated website as a ‘one-stop portal’ should therefore be considered for maintaining transparency and sufficient circumstantiality. Such a portal would be complete with a user-oriented guide and be connected with a single national repository of ‘authorized’ information. This would maintain the standardized quality of and ease of accessibility to information for audiences with varying IT skills. ‘Official’ drug information storage at a single national repository might facilitate the transparency of drug information at the population level, such as in media publicity, and might also satisfy individual patient needs during consultation at a doctor’s office, as well as in pharmacological training at dispensing pharmacies.

## Electronic supplementary material

Additional file 1: **Outline of the survey questionnaire (English translation).** (DOC 49 KB)

Additional file 2: **Media Doctor Australia rating instrument for Adverse Effects.** (DOC 50 KB)
