# Supplementary material for: Pharmaceutical company perspectives on current safety risk communications in Japan
Source: Springerplus. 2014 Jan 24;3:51. doi: 10.1186/2193-1801-3-51 (PMC3921345; doi:10.1186/2193-1801-3-51)
Supplement: Supplementary file 1 — Additional file 1: Outline of the survey questionnaire (English translation). (DOC 49 KB) [file 40064_2013_806_MOESM1_ESM.doc]

**Online Resource 1**

Outline of the survey questionnaire (English translation).

I．Contents for risk communication

1. Please prioritize the following aspects when transmitting risk messages regarding drug safety?

Strength of evidence, Stratification of patients with regard to harm, Number of people affected by harm, Novelty of harm, Quantification of harms, Benefit to harm ratio, Sources of information, and Treatment options.

1. What do you pay attention to in creating messages for risk communication, including company policies? (open question)
2. What do you pay attention to in conveying messages for risk communication, including company policies? (open question)

II．Targets for risk communication

1. Please prioritize the following risk communication targets.

Patients, Public, Physicians, Pharmacists, Other paramedics, Regulatory bodies, In-company divisions, and Mass media.

1. Please rate your degree of success in risk communication with each of the above targets.
2. Please give percentage estimates of workload allocation of your pharmacovigilance department to each risk communication target.

III．Measures used for risk communication

1. Has your company developed Drug Guides for Patients?
2. Do you think the Drug Guides for Patients are useful for patients? Please give the reason.
3. Please rate the presentation of Drug Guides for Patients posted on the Pharmaceuticals and Medical Devices Information Homepage, according to the following criteria.

Easy to read, Easy to find what I need, Organization of information, Font and font size, Length, Relevance to your specialty of area of expertise, Understandable, Timeliness, Helpful, and Amount of background information.

1. Do you think that that the Drug Guides for Patients is to be developed for all the prescription drugs is useful for patients? Please give the reason.
2. Do you think that risk communications via the websites run by your company are useful for patients? Please give the reason.
3. Do you think that risk communications via the websites run by your company is useful for healthcare professionals? Please give the reason.
4. Do you think the explanation materials of package insert revisions are useful for the public and patients, if any? Please give the reason.
5. Please give your comment on the improving of safety information provided by the regulatory bodies, if any. (open question)
6. Please identify three effective measures in emergency risk communications and rank them in their order of effectiveness.

Regulatory measures including a Dear Healthcare Professional letter and revision of a package insert, Direct communication by the regulatory authority, Direct communication by company medical representatives, Publication via mass media or academic journals, and Communication via companies’ websites

1. Do you think disclosure of risk management plan is useful? Please give your comments, if any.

Article title: Pharmaceutical company perspectives on current safety risk communications in Japan.

Journal: SpringerPlus

Authors: Hisashi Urushihara, Gen Kobashi, Hideaki Masuda, Taneichi Setsuko, Michiko Yamamoto, Takeo Nakayama, Koji Kawakami, Tsutomu Matsuda, Kaori Ohta, Hiroki Sugimori

Corresponding to: Hiroki Sugimori

Graduate School of Sports and Health Science

Department of Preventive Medicine

Daito Bunka University

E-mail: hsugimor@ic.daito.ac.jp
