# Supplementary material for: Pharmaceutical company perspectives on current safety risk communications in Japan
Source: Springerplus. 2014 Jan 24;3:51. doi: 10.1186/2193-1801-3-51 (PMC3921345; doi:10.1186/2193-1801-3-51)
Supplement: Supplementary file 2 — Additional file 2: Media Doctor Australia rating instrument for Adverse Effects. (DOC 50 KB) [file 40064_2013_806_MOESM2_ESM.doc]

**Online Resource 2**

Media Doctor Australia rating instrument for Adverse Effects.

| **Rating Criteria** | **Satisfactory** | **Not Satisfactory** |
| --- | --- | --- |
| Benefit to harm ratio | Tries to balance reporting of both benefits and harms or gives some sense of the ratio between the two. | No mention of whether therapy has more benefits or more harms. |
| Novelty of harm | Mentions whether or not harm was previously identified or mentions what is added to previous knowledge about harm. | No mention of whether or not harm has previously been recognised. |
| Evidence | Where relevant there is mention of strength of evidence and correct interpretation. | No mention of the nature of clinical research that lead to recognition of harm or increased frequency of harm. |
| Quantification of harms | Even some quantification of harm rates an 'S'. | No quantification of harm or describes it using words such as 'minor' or 'not serious'. |
| Number of people affected by harm | Some quantification of number of people or percent of people affected by the harm. | No mention of numbers or percent of people taking treatment expected to be harmed. |
| Stratification of patients with regard to harm. | Mentions which groups of patients are most likely to be harmed. | No mention of which groups of patients are most likely to suffer harm. |
| Sources of information | Provides detail on information sources and their potential COI, and reports independent source or mentions unsuccessful attempt to obtain corroboration. | No mention of sources or possible conflicts of interest. No attempt at independent corroboration. |
| Treatment options | Mentions alternatives and discusses whether alternatives are more or less harmful. | No mention of alternatives or whether the alternatives are more or less harmful. |
| Relies on Press Release | No obvious use of text from the press release. | Evidence from press release or other news storie that the journalist has relied on a press release as the only information source and used the text in the story. |

Available from <http://www.mediadoctor.org.au/content/ratinginformation.jsp>.

Permission to use this instrument was obtained from Media Doctor Australia.

Article title: Pharmaceutical company perspectives on current safety risk communications in Japan.

Journal: SpringerPlus

Authors: Hisashi Urushihara, Gen Kobashi, Hideaki Masuda, Taneichi Setsuko, Michiko Yamamoto, Takeo Nakayama, Koji Kawakami, Tsutomu Matsuda, Kaori Ohta, Hiroki Sugimori

Corresponding to: Hiroki Sugimori

Graduate School of Sports and Health Science

Department of Preventive Medicine

Daito Bunka University

E-mail: hsugimor@ic.daito.ac.jp
